# Refractory immune thrombocytopenia responding to combination therapy of eltrombopag and low-dose rituximab: a case series

**DOI:** 10.1016/j.htct.2024.03.011

**Published:** 2024-08-18

**Authors:** Tan-Huy Chu, Thien-Ngon Huynh, Quoc-Vu Trinh-Le, Chi-Dung Phu

**Affiliations:** a Adult hematology department no.2, Blood Transfusion Hematology Hospital, Ho Chi Minh City, Vietnam; b Department of Hematology, Pham Ngoc Thach University of Medicine, Ho Chi Minh City, Vietnam; c Director Board, Blood Transfusion Hematology Hospital, Ho Chi Minh City, Vietnam

## Introduction

**Figure 1:**
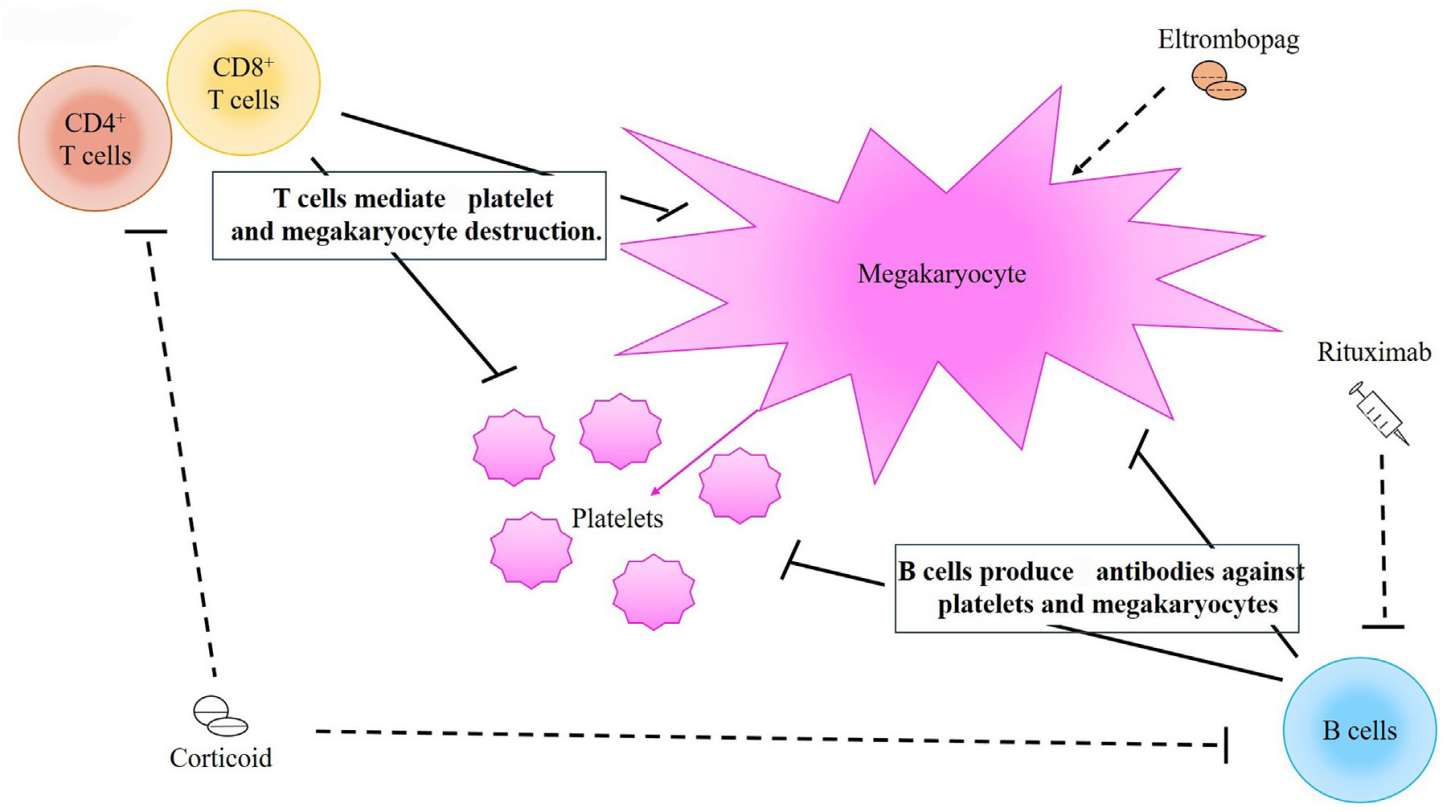
Pathophysiology of immune thrombocytopenia, with the key event being the production of anti-platelet antibodies by B cells. These antibodies destroy platelets and megakaryocytes via the complement system and macrophages. The other mechanism involves CD4^+^ and CD8^+^ T cells, which directly destroy and inhibit the production of platelets. Glucocorticoids inhibit the proliferation of B cells and T cells and rituximab targets and marks B cells for destruction. Eltrombopag stimulates the production of new platelets. The combination therapy of eltrombopag with rituximab involves both promoting platelet production and strongly inhibiting platelet destruction.

Immune thrombocytopenia (ITP) is an autoimmune disease, characterized by thrombocytopenia. With a low platelet count, the patients may present without symptoms to mild mucocutaneous hemorrhages, or even life-threatening bleeding. However, the overall risk (only around 5 %) of severe bleeding is low. Overall, ITP is a common disease with around four cases per 100.000 person-years. The pathophysiology of ITP is complex, with the main two mechanisms involving autoantibodies inducing the destruction of platelets and inhibition of megakaryocyte production and function. Nevertheless, autoantibodies against platelets are not detected in up to 50 % of ITP patients, which raises the likelihood of an alternative mechanism, involving increased production and reaction of T helper 1 (Th1) cells and T helper 17 (Th17) cells, along with reduce production and function of regulatory T cells (Figure 1).^1^, ^2^, ^3^

ITP diagnosis is defined in patients with a platelet count below 100 × 10^3^/µL, when other causes of thrombocytopenia have been ruled out^1^^,^^4^. Glucocorticoids are the standard initial regimen for patients with ITP, with 60–80 % of ITP patients having an initial response, however, only 30–50 % have a prolonged response after glucocorticoids are suspended. The common medical treatments for ITP patients who failed with glucocorticoids are thrombopoietin receptor agonists (eltrombopag), immunomodulators (rituximab), and splenectomy. Eltrombopag is the most recommended after glucocorticoids have failed, with response achieved in 40–60 % of patients with continuous therapy^1^^,^^5^. However, when eltrombopag fails, treatment has come to a standstill.

Here, we report our experiences of using combination therapy with eltrombopag and low-dose rituximab on four refractory ITP patients, who failed first-line treatment of methylprednisolone and second-line treatment of eltrombopag.

## Case presentations

**Figure 2:**
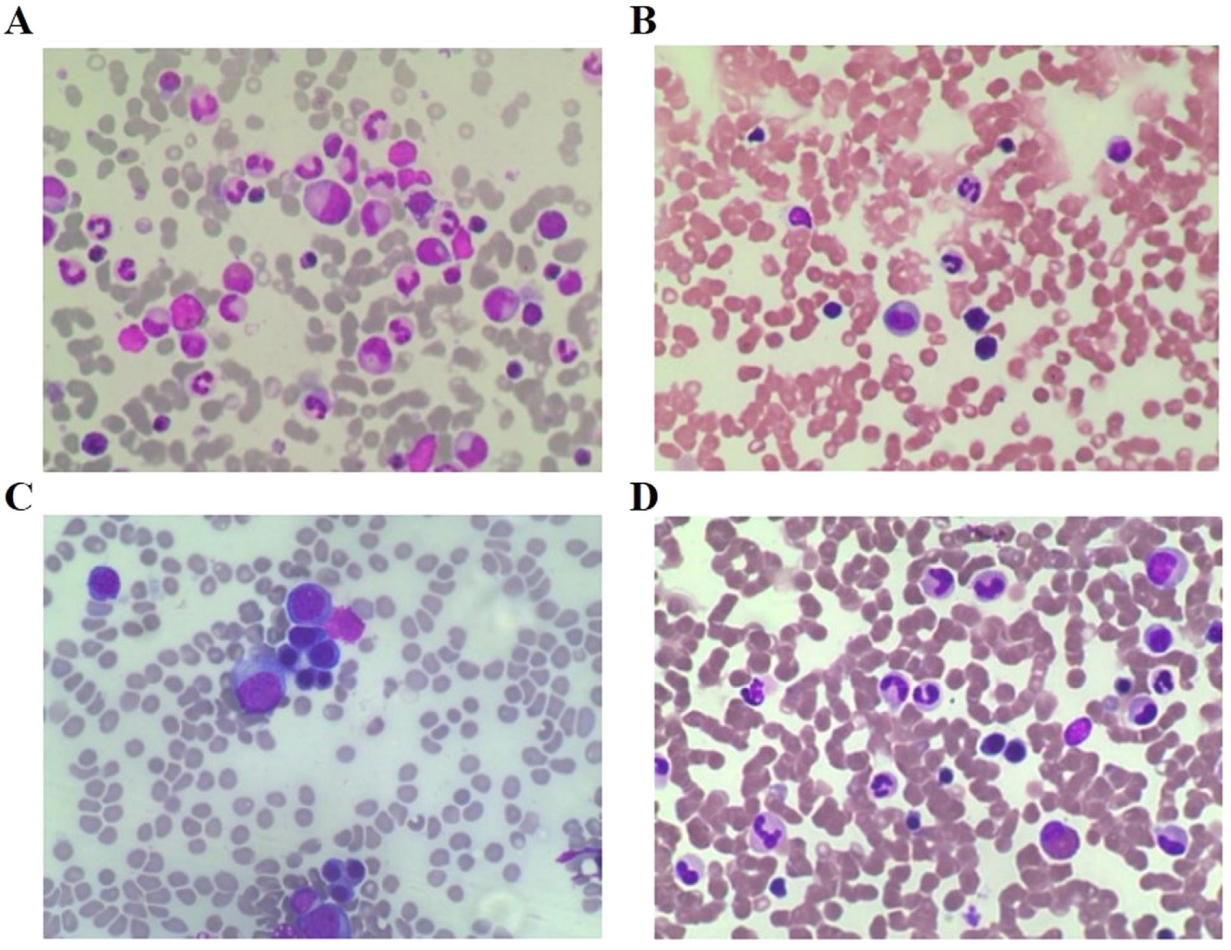
The bone marrow aspiration at the time of hospital admission, in A) Case 1 B) Case 2 C) Case 3 and D) Case 4. The images show no abnormalities.

**Figure 3:**
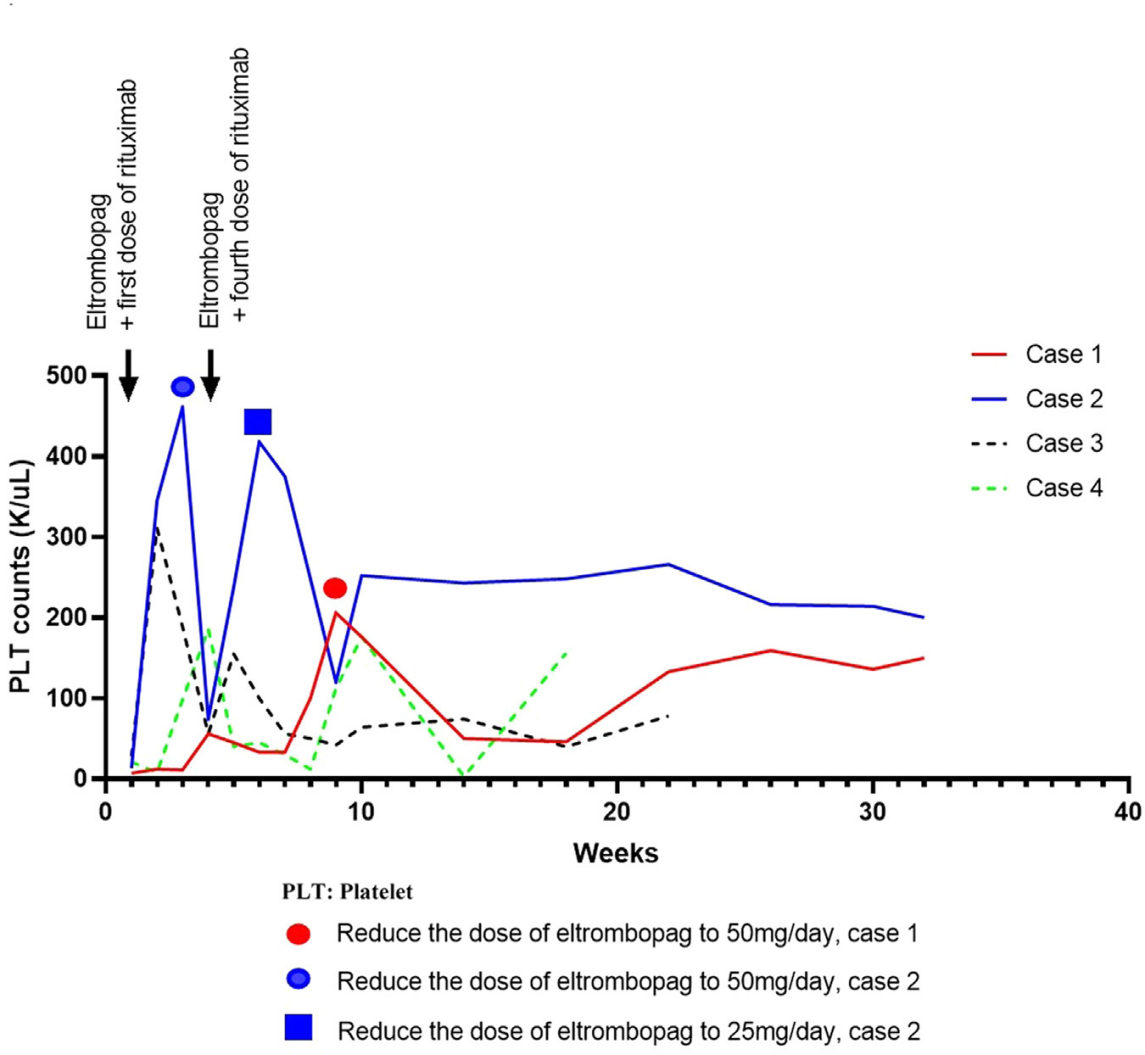
Platelet counts of all cases at the time of treatment and follow-up.

In this report, we retrospectively reviewed the clinical data of four patients, who were admitted to Blood Transfusion Hematology Hospital, Vietnam. The patients had the following characteristics: (1) diagnosis with ITP for over three months, (2) refractory to first-line treatment of methylprednisolone (1 mg/kg/day) and second-line treatment of eltrombopag (75 mg/day) for at least two consecutive weeks, and (3) negative for ANA, anti-dsDNA, anti-SM, HbsAg, HCV, HIV, with bone marrow aspiration showing no abnormalities (Figure 2). All patients in this study underwent a combination therapy of oral eltrombopag (75 mg/day) and intravenous rituximab (100 mg/week × 4 weeks). The rituximab dose was chosen based on a previous study^6^. When the platelets reached over 30 × 10^3^/µL for one week with no bleeding then response to therapy was characterized and when platelets reached over 100 × 10^3^/µL for one week without bleeding it was considered as a complete response. The eltrombopag dose was adjusted based on platelet count according to the manufacturer's instructions. In all four cases, the patients responded to the combination treatment, with no noticeable adverse effects. Patient demographics and laboratory results are presented in Tables 1 & 2 and Figures 2 & 3. A detailed summary of all cases is as follows:

**Table 1:** Clinical characteristics of the immune thrombocytopenia (ITP) patients.

Characteristic	Case 1	Case 2	Case 3	Case 4
Gender	Female	Female	Female	Male
Age (years)	23	65	66	64
Presenting symptoms	scattered hemorrhage, menorrhagia	scattered hemorrhage	scattered hemorrhage	scattered hemorrhage
Comorbidities	none	hypertension, type 2 diabetes	hypertension, type 2 diabetes	none
Follow-up time (weeks)	32	32	21	18
From ITP diagnosis to combination treatment	5 years	7 months	12 years	3 years
Time to first response after first rituximab dose (weeks)	4	3	2	3
Eltrombopag dose to maintain response (mg/day)	50	25	75	75

**Table 2:** Laboratory characteristics of the immune thrombocytopenia patients before and after 4 weeks of combination treatment.

	Case 1		Case 2		Case 3		Case 4	
Characteristic	*Before*	*After*	*Before*	*After*	*Before*	*After*	*Before*	*After*
Hemoglobin (g/dL)	9.3	9.3	12.7	13.5	14.8	14.8	13.8	15.3
Platelet count (x 10^3^/µL)	7	56	13	74	28	56	21	186
White blood cells (x 103/µL)	6.4	5.1	9.3	10.5	9.7	9	8.5	12
Aspartate transaminase (AST) (U/L)	28	19	17	19	56	62	15	23
Alanine transaminase (ALT) (U/L)	10	18	16	19	22	29	15	18
Direct/Total bilirubin (µmol/L)	1.5/10	1.2/11	1.2/26	1.4/18	2/19.6	1.4/14.7	1/7.4	1.7/14.7
Creatinine (µmol/L)	60.7	64	73	89	76	80	71	86

AST: aspartate aminotransferase; ALT: alanine aminotransferase

## Case 1

A 23-year-old female patient with ITP over five years was treated with methylprednisolone but suffered many relapses. Five months before hospital admission, the patient was diagnosed with chronic ITP however, she failed methylprednisolone treatment and so she received eltrombopag (50 mg/day) and methylprednisolone (8 mg/day) over four months. As her platelet count remained below 10 × 10^3^/µL the eltrombopag dose was eventually increased to 75 mg/day. Nevertheless, her platelet count did not respond during two weeks of treatment. When admitted to hospital, she was experiencing scattered hemorrhage with menorrhagia. The laboratory test results are shown in Table 1. She was diagnosed with chronic ITP refractory to glucocorticoid and eltrombopag treatment and the regimen was changed to eltrombopag and low-dose rituximab. Her platelet count had responded by the fourth rituximab dose, reaching 56 × 10^3^/µL. Nine weeks after the first rituximab dose, her platelet count had reached 206 × 10^3^/µL, and the dose of eltrombopag was reduced to 50 mg/day. After 32 weeks of follow-up, she still remains in complete response (Figure 3).

## Case 2

A 65-year-old female patient had been diagnosed with ITP seven months previous to hospitalization when she was treated with methylprednisolone for three months. Nevertheless, her platelet count remained below 10 × 10^3^/µL. She then suffered a severe headache and was diagnosed with intracranial hemorrhage. At that time, she was treated with platelet transfusions and intravenous immunoglobulin (IVIG - 1 g/kg/day) for two days and her platelet count reached 56 × 10^3^/µL for two days then it decreased to below 10 × 10^3^/µL. Four months before hospital admission, the patient was diagnosed with persistent ITP and failed methylprednisolone treatment so she was treated with eltrombopag (50 mg/day) and methylprednisolone (8 mg/day) for two months. Her platelet count remained below 30 × 10^3^/µL and eventually the eltrombopag dose increased to 75 mg/day. Nevertheless, after another two months of treatment, she still had not responded. When admitted to hospital, she was experiencing scattered hemorrhage. The laboratory test results are shown in Table 1. She was diagnosed with persistent ITP refractory to glucocorticoid and eltrombopag treatment. She was treated with eltrombopag and low-dose rituximab. Her platelet count reached 316 × 10^3^/µL after the second rituximab dose. With the third rituximab dose, her platelet count reached 461 × 10^3^/µL and so the dose of eltrombopag was reduced to 50 mg/day and subsequently to 25 mg/day. After 32 weeks of follow-up, she maintained a complete response (Figure 3).

## Case 3

A 66-year-old female patient was diagnosed with ITP 12 years prior to hospitalization; she was treated with methylprednisolone but suffered many relapses. Three months before hospital admission, the methylprednisolone treatment failed and she was diagnosed with chronic ITP, a condition treated with eltrombopag (50 mg/day) and methylprednisolone (8 mg/day) over two months. As her platelet count remained below 30 × 10^3^/µL, the eltrombopag dose was increased to 75 mg/day, however one month later her platelet count still had not responded. When admitted to hospital, she was experiencing scattered hemorrhage. The laboratory test results are shown in Table 1. She was diagnosed with chronic ITP refractory to glucocorticoid and eltrombopag treatment so the regimen was changed to eltrombopag and low-dose rituximab. The first response was seen after the second dose of rituximab and at the third rituximab dose her platelet count reached 315 × 10^3^/µL. However, at the fourth rituximab dose her platelet count had dropped to 56 × 10^3^/µL. Over 22 weeks of follow-up, her platelet count ranged from 42 to 78 × 10^3^/µL (Figure 3).

## Case 4

A 64-year-old male patient was diagnosed with ITP three years prior to hospitalization; he was treated with methylprednisolone but suffered many relapses. Twelve months before hospital admission, the patient had failed methylprednisolone treatment and was diagnosed with chronic ITP and accordingly he was treated with eltrombopag (50 mg/day) and methylprednisolone (8 mg/day) for 10 months. As his platelet count remained below 40 × 10^3^/µL, the eltrombopag dose was increased to 75 mg/day. However, after two months of treatment his platelet count still had not responded. When admitted to hospital, he was experiencing scattered hemorrhage. The laboratory test results are shown in Table 1. He was diagnosed with chronic ITP refractory to glucocorticoid and eltrombopag treatment. He was treated with eltrombopag and low-dose rituximab. His platelet count reached 98 × 10^3^/µL by the third rituximab dose and 186 × 10^3^/µL by the fourth rituximab dose. Over 18 weeks of follow-up, his platelet count ranged from 34 to 176 × 10^3^/µL (Figure 3).

## Discussion

When first-line methylprednisolone treatment and second-line eltrombopag treatment fail, the treatment comes to a standstill with the only options left being splenectomy and immunomodulatory drugs. Splenectomy can induce long-lasting remission in 60 % of ITP patients, however, splenectomy has become less used due to post-operative complications such as venous thromboembolism and sepsis. Splenectomy is also generally not performed in frail elderly ITP patients due to the increased risk of complications^1^. In addition, other immunomodulatory drugs such as mycophenolate mofetil, azathioprine, dapsone, and danazol are used in ITP patients with limited supporting data and low response rates.^1^^,^^7^

The present study was conducted to overcome this problem in refractory ITP patients. To the best of our knowledge, we are the first group to report the use of combination therapy using eltrombopag and low-dose rituximab in refractory ITP patients who failed methylprednisolone and eltrombopag treatment. Rituximab, a monoclonal antibody against CD20, is the most frequently prescribed antibody for ITP patients however, it is not approved by the FDA for this indication. Rituximab gave a 60 % initial response rate in ITP patients, and 20–30 % after two years, with a median time to response (TTR) of 5.5 weeks.^1^^,^^6^ In a previous study, Hai Zhou et al. reported that using rituximab with recombinant human thrombopoietin compared to rituximab monotherapy in ITP patients who had failed glucocorticoid therapy, showed a significantly shorter TTR (7 days versus 28 days) and higher complete response rate (45.4 % versus 23.7 %). However, there is no significant difference in the overall response rates (79.2 % versus 71.1 %). Similar to Hai Zhou et al., in the current study ITP patients have similar TTR (median: 3; range: 2–4 weeks); all four patients had a response to combination therapy with two patients achieving complete responses.

In the present study, we noticed that in the second case, the patient had been treated for ITP over seven months, and achieved a complete response with the combination therapy even after reducing the eltrombopag dose to 25 mg/day. In contrast, in the third case, the patient was diagnosed with ITP 12 years previously and only achieved response with the combination therapy without reducing the eltrombopag dose. Thus, we hypothesize that the shorter duration of ITP may contribute to the response of the patient. Previous studies also supported this hypothesis with rituximab treatment in ITP patients showing that younger patients, female gender, and shorter duration of ITP might be factors for better outcomes.^6^^,^^8^^,^^9^ A rapid increase and high response rate in platelet count in this study was beneficial for refractory ITP patients. This result suggests a synergistic mechanism between eltrombopag and low-dose rituximab with both promoting platelet production and strongly inhibiting platelet destruction.^1^^,^^6^^,^^8^^,^^10^

In conclusion, we would like to share our experience in treating refractory ITP patients, who failed methylprednisolone and eltrombopag therapy when treatment usually comes to a standstill. The combination therapy of eltrombopag and low-dose rituximab has a high response rate and rapid increase in the platelet count in refractory ITP patients. Furthermore, the use of combination therapy with a shorter duration of ITP can contribute to better outcomes. We hope that this study will assist other physicians in selecting the appropriate treatment in such situations.

## Ethical approval

This study was approved by our hospital Institutional Review Board.

## Informed consent

The written consent form was acquired from the patient prior to the study.

## Consent for publication

The written consent form was acquired from the patient prior to the study.

## Conflicts of interest

All authors have declared that there are no financial conflicts of interest with regard to this work.
